# Advances in salmonid genetics—Insights from Coastwide and beyond

**DOI:** 10.1111/eva.13732

**Published:** 2024-06-17

**Authors:** Shawn R. Narum, Matthew Campbell, Katharine Coykendall, Mariah Meek, Kathleen G. O'Malley, Maren Wellenreuther

**Affiliations:** ^1^ Hagerman Genetics Laboratory Columbia River Inter‐Tribal Fish Commission Hagerman Idaho USA; ^2^ Idaho Department of Fish and Game Eagle Fish Genetics Lab Eagle Idaho USA; ^3^ Eagle Fish Genetics Lab Pacific States Marine Fisheries Commission Eagle Idaho USA; ^4^ Department of Integrative Biology Michigan State University, the Wilderness Society Bozeman Montana USA; ^5^ State Fisheries Genomics Lab, Coastal Oregon Marine Experiment Station, Department of Fisheries, Wildlife and Conservation Sciences, Hatfield Marine Science Center Oregon State University Newport Oregon USA; ^6^ The New Zealand Institute for Plant and Food Research Limited, Seafood Production Nelson New Zealand; ^7^ School of Biological Sciences University of Auckland Auckland New Zealand

**Keywords:** conservation genetics, fisheries management, life history evolution

## Abstract

This article summarizes the Special Issue of Evolutionary Applications focused on “Advances in Salmonid Genetics.” Contributions to this Special Issue were primarily presented at the Coastwide Salmonid Genetics Meeting, held in Boise, ID in June 2023, with a focus on Pacific salmonids of the west coast region of North America. Contributions from other regions of the globe are also included and further convey the importance of various salmonid species across the world. This Special Issue is comprised of 22 articles that together illustrate major advances in genetic and genomic tools to address fundamental and applied questions for natural populations of salmonids, ranging from mixed‐stock analyses, to conservation of genetic diversity, to adaptation to local environments. These studies provide valuable insight for molecular ecologists since salmonid systems offer a window into evolutionary applications that parallel conservation efforts relevant and applicable beyond salmonid species. Here, we provide an introduction and a synopsis of articles in this Special Issue, along with future directions in this field. We present this Special Issue in honor of Fred Utter, a founder and leader in the field of salmonid genetics, who passed away in 2023.

## 1 INTRODUCTION

Salmonid fishes have been heavily studied and monitored for many years due to their extensive ecological, cultural, and economic importance throughout the world. Across the west coast of North America, Pacific salmonids are a vital component of the ecosystem, providing benefits to all trophic levels in aquatic and riparian habitats. They have evolved over millions of years under stochastic geological events (Lindsey & McPhail, 1986; Waples et al., 2008) and have adapted to local environments while also colonizing newly available stream systems (Quinn, 2005; Taylor, 1991). These salmonid species exhibit highly diverse life histories that have illuminated a wide variety of evolutionary processes (e.g., Hendry & Stearns, 2004), yet much remains unknown and waiting to be discovered. For several decades, genetic markers in salmonids have been applied to enhance research into life history variation, population structure, and mixed‐stock fisheries (Allendorf & Utter, 1979; Utter et al., 1973), but rapid advances in technology have brought genomic tools to the forefront of discovery in recent years. Thus, this Special Issue of Evolutionary Applications focuses on recent advances and contributions to our understanding of salmonids through genetic and genomic analyses.

**FIGURE 1:**
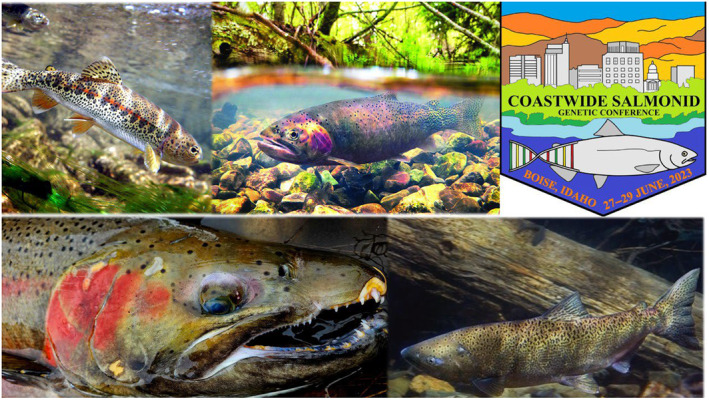
Collage of Pacific salmonids (*Oncorhynchus*) representing a portion of the study species in this Special Issue. Clockwise from top left, Redband Trout (*O. mykiss gairdneri*), hybrid Cutthroat Trout (*O. clarkii*), Coastwide Salmonid Genetics Meeting logo, Chinook Salmon (*O. tshawytscha*), and Steelhead (*O. mykiss*).

The articles in this Special Issue represent 22 studies with a wide range of topics including mixed‐stock analyses, parentage‐based tagging applications, conservation of life history variation and genetic diversity, adaptive genomic variation, and anthropogenic effects on salmonid genomes (Figure 1). The majority of articles represent contributions from the Coastwide Salmonid Genetics Meeting (“Coastwide”) that was held in Boise, ID, in June 2023, with a focus on Pacific salmonids of the west coast of North America. This meeting has persisted for decades, with a modest start in the 1980s, and with a focus on mixed‐stock applications, but has continued to grow and expand to a wide variety of topics (see Teel et al., 2011 for more history of the Coastwide meeting). After a hiatus due to the global coronavirus pandemic, Coastwide resumed in 2023 with an outpouring of major advances that had been pent up in the genetics community. We provide a synopsis of these advances in the sections below, along with anticipated future advances in the field of salmonid genetics/genomics.

## 2 SYNOPSIS OF ARTICLES—ADVANCES IN SALMONID GENETICS

### 2.1 Mixed‐stock analyses and parentage‐based tagging

The following studies highlight the evolving nature of genetic tools and methodologies in fisheries science involving genetic stock identification (GSI), mark–recapture methods, species identification, and parentage‐based tagging (PBT). They collectively contribute to our understanding of salmonid species' genetic dynamics, while offering practical tools for sustainable fisheries management and conservation.

Hargrove et al. (2024) explored the optimization of a GSI baseline aimed at Steelhead *Oncorhynchus mykiss* conservation and fisheries management in the Snake River Basin in the northwest United States. The study compared different genetic baseline configurations involving sample sizes and population representation, marker numbers, and marker types to assess their impact on the accuracy of stock assignments. Results indicated that a newly developed baseline with reduced sample sizes, but more representative sampling, and an increased number of genetic markers, significantly improved self‐assignment rates without increasing bias. This study should aid other GSI efforts aimed at reducing baseline genotyping efforts and costs while improving GSI accuracy.

Hsu and Habicht (2024) introduced a novel method for GSI called the “integrated multistage framework” (Ms.GSI), designed to enhance the resolution of GSI analyses in mixed‐stock fisheries. Traditionally, GSI is conducted regionally, limiting its applicability to broad geographic ranges due to non‐overlapping populations and genetic markers. The Ms.GSI framework overcomes this limitation by integrating disparate regional baselines into a single process, enabling accurate estimation of stock contributions across wide areas. Unlike the conventional two‐step approach, which suffers from misassignments and underestimation of uncertainty, Ms.GSI produced more reliable estimates and less error propagation. However, biases such as reporting‐unit bias remain challenging. Nonetheless, Ms.GSI offers a more efficient and straightforward alternative, potentially reducing costs and time in baseline development and facilitating collaboration among research institutions across regions.

Hess et al. (2024) explored the integration of GSI into test fisheries to improve the management of Columbia River Spring Chinook Salmon *O. tshawytscha*. Over five years, the study assessed how GSI can be refined for stock‐specific run timing and abundance predictions when used alongside visual stock identification (VSI). While VSI was able to effectively forecast broad stock categories, GSI/PBT provided more detailed predictions down to hatchery broodstock levels, but faces some challenges with natural‐origin stocks due to limited sampling. The test fishery data combined with GSI/PBT offer valuable insights for fisheries planning, aiding in the prediction of upriver stock abundance and timing. Despite the promise of this approach, the study acknowledges logistical hurdles in swiftly processing genetic samples and converting catch per unit effort (CPUE) into accurate adult salmon counts. These findings suggest that integrating GSI/PBT with VSI in test fisheries can enhance fisheries management but emphasize the need for ongoing refinement to accommodate variability in salmon behavior and environmental conditions, ultimately contributing to more sustainable management of the Chinook Salmon populations in the Columbia River.

Rosenbaum et al. (2024) investigated the efficacy of transgenerational genetic mark–recapture (tGMR) as a tool for estimating population abundance, particularly focusing on Chilkat River Chinook Salmon in Southeast Alaska. Their study compared tGMR estimates with traditional mark–recapture methods and explored the impact of adult sampling location and timing on tGMR accuracy. Results showed that tGMR estimates using a representative sample of mainstem adults closely aligned with traditional estimates and offered slightly higher precision. However, the study highlighted the influence of adult sampling location and timing on abundance estimates, emphasizing the need for careful sampling design. Moreover, simulations identified potential biases in tGMR estimates, particularly related to variations in reproductive success and sampling selectivity. Despite these challenges, tGMR was validated as a promising tool for population enumeration, offering increased precision and reduced handling stress for adult spawners compared to traditional methods. The study underscores the importance of considering system‐specific sampling designs and provides valuable insights into addressing key assumptions and biases associated with tGMR applications.

Robinson et al. (2024) focused on improving the misidentification of salmonid species in large‐scale genotyping efforts. To address this, the study identified 19 primer pairs with 51 species‐informative variants, facilitating discrimination among 11 salmonid species, including two Cutthroat Trout subspecies. These markers were incorporated into existing genotyping panels for Chinook Salmon, Coho Salmon, Sockeye Salmon, and Steelhead, enhancing species identification accuracy. A species‐calling script was developed to automate the identification of non‐target species and was proven effective through empirical and simulated data tests. This approach, utilizing existing amplicons for species discrimination, offers a cost‐effective solution for genetic monitoring programs, aiding Pacific salmon conservation efforts in the Columbia River basin and potentially beyond.

Goetz et al. (2024) focused on understanding how genetic lineages and environmental factors influence variation of life history traits in steelhead in California. Researchers genotyped over 23,000 Steelhead returning to four California Central Valley (CCV) hatcheries from 2011 to 2019. Based on PBT pedigrees, they found significant differences in adult life history traits among Steelhead from different genetic lineages, despite inhabiting similar environments. Coastal and Central Valley Steelhead lineages showed differences in age at return, timing of spawning, and rates of iteroparity. Additionally, adaptive genetic variation associated with migratory life history traits varied among hatchery programs, with the Omy05 chromosome showing distinct patterns, especially in coastal‐origin steelhead. The study suggests that different Steelhead lineages may respond differently to changing environments since each lineage maintains distinct phenotypic and genetic variation over generations. This research provides valuable insights into the complex interactions between genetics, environment, and management strategies in shaping the life history of Steelhead populations.

Horn et al. (2024) explored the reintroduction of Coho Salmon (*O. kisutch*) to the interior Columbia River, following their local extinction in the mid‐1900s. The success of these reintroduction efforts has largely been credited to the work of the Confederated Tribes of the Umatilla Indian Reservation, the Confederated Tribes and Bands of the Yakama Nation, and the Nez Perce Tribe. A key milestone in this process was the development of a PBT baseline for Coho Salmon from hatcheries within the interior Columbia River basin and adjacent areas. This baseline, encompassing over 32,000 samples from nine hatcheries across 9 years, allowed for a range of uses, including tracing the origin of fish in mixed‐stock fisheries and evaluating genetic diversity within various hatchery programs. The PBT baseline has proved instrumental in locating hatchery‐origin fish on spawning grounds and understanding the composition of mixed‐stocks passing dams like Priest Rapids. The PBT baselines, accessible via FishGen.net, support continuous management and reintroduction activities by providing insights into population genetics, dispersal patterns, and the overall sustainability of restored Coho Salmon. Furthermore, the PBT baseline opens up new avenues for evaluating reintroduction success and can serve as a guide for future strategies aimed at maintaining genetic diversity and avoiding inbreeding.

### 2.2 Conservation of life history variation and genetic diversity

Several articles in this Special Issue investigated effects of anthropogenic disturbances on diversity within and among salmonid species. Conservation of diversity is particularly important for salmonids that display a wide variety of life history variation across environments that enable them to persist and adapt over evolutionary time. Two studies investigated the effects of hatchery programs on diversity (McPhee et al., 2024) and fitness (Dayan et al., 2024) of natural populations, while multiple studies examined effects to diversity of altered habitats (Hugentobler et al., 2024; Moccetti et al., 2024) or harvest impacts (Miettinen et al., 2024). Two studies characterized variation at the genomic (Carbonneau et al., 2024) or transcriptomic (Kokkonen et al., 2024) level to assist with conservation of rare taxa and remote populations, while Thompson et al. (2024) used genomic study to better understand life history diversity of imperiled populations. Finally, Strait et al. (2024) examined effects of natural environments and introgression on cardiac performance of *O. clarkii* under heat stress.

McPhee et al. (2024) investigated effects of a supplementation program for Sockeye Salmon (*O. nerka*) in Auke Lake, Alaska. Parentage analyses of returning adults indicated that the program provided a demographic boost in productivity in the 3 years evaluated (ranging from 6.0–48.6 times higher productivity), presumably due to limited spawning habitat and density dependence during incubation stages for fish in natural habitat. There was no statistical difference in adult run timing or size at age between hatchery reared versus wild fish, but hatchery‐reared fish were younger as returning adults on average than wild fish. Despite that a portion of wild adults returned after only one year in the ocean (~2%), significantly more hatchery‐reared fish returned after one year in freshwater than wild fish, leading to overall younger age of hatchery‐reared fish. High growth rates during hatchery rearing may have led to younger age at maturity as is commonly observed in hatchery programs for salmonids. However, this rearing effect on age at maturity was not evaluated through subsequent generations when hatchery fish spawned in nature to determine if there were subsequent effects to the wild population.

Dayan et al. (2024) investigated whether low reproductive success of hatchery‐origin Chinook Salmon (*O. tshawytscha*), compared to natural‐origin Chinook Salmon, persisted among their wild‐born descendants. To address this, they genotyped nearly 10,000 fish over 13 years and used genetic parentage analysis to compare the reproductive success of three groups of adult Chinook Salmon reintroduced above Cougar Dam on the South Fork McKenzie River, Oregon. The three groups included (1) hatchery‐origin (HOR) Chinook Salmon from an integrated stock, (2) first‐generation, wild‐born descendants (F_1_s) of Chinook Salmon produced at the same hatchery and (3) natural‐origin (NOR) Chinook Salmon that were presumed to have been produced below the dam. They found that F_1_s produced significantly more adult offspring than HOR Chinook Salmon that spawned alongside them in the same river. In addition, the F_1_s produced nearly as many adult offspring as the NOR fish. This finding suggests that, for the South Fork McKenzie reintroduction program, a single generation in the wild increased fitness for descendants of HOR Chinook Salmon. The researchers clearly outlined important limitations to their findings that must be considered before applying their conclusions to other reintroductions, hatchery supplementation programs, hatchery augmentation programs, or hatchery risk evaluations.

Moccetti et al. (2024) applied a before‐and‐after control‐impact (BACI) study to quantify the effect of a fish pass system on genetic diversity and differentiation in Brown Trout (*Salmo trutta*) populations. The authors used 81 SNPs to examine the patterns of *H*_*o*_, *H*_*S*_, *F*_*ST*_, *F*_*IS*_, gene flow, and admixture in tributaries before and after a fish pass system was installed in one of the tributaries. Somewhat surprisingly, they found differentiation between formerly disjunct populations increased after fish pass installation. Citing telemetry data from the same system, the authors posited that the fish pass system facilitated homing to upstream spawning grounds in Brown Trout that were previously excluded from upstream habitat by a weir. However, in their control system lacking migratory barriers, genetic homogeneity was observed suggesting a lack of homing behavior. This study illustrates that genetic tools are useful for monitoring the effects of habitat fragmentation and mitigations and shifting genetic patterns can be observed soon after habitat modifications are installed.

Hugentobler et al. (2024) explored the detailed relationship between arrival timing of Chinook Salmon on spawning grounds and genotypes at the GREB1L region of the genome in the Central Valley of California. They combined tagging data on freshwater arrival data and passage date to spawning habitat upriver with GREB1L genotypes to determine whether spring‐run Chinook Salmon are still present in the highly impacted Yuba River. They show that there is indeed both genotypically and phenotypically spring run in the system, which is important for protecting this imperiled ESU and to promote a healthy portfolio of spring‐run populations in the Central Valley. Interestingly, their results find that fish that are homozygous for the early migrating alleles and those that are heterozygous show up lower in the system during the same time period; however, they move onto the spawning grounds at different times. Those that are homozygous early moving into the spawning grounds first, followed by those that are heterozygous, and those that are homozygous for the late alleles spawning latest. These results are highly promising for the conservation of the spring‐run phenotype in the Central Valley as it shows preservation of these phenotypes and genotypes for Chinook Salmon even in a highly impacted system such as the Yuba River.

Thompson et al. (2024) also used genomic study to investigate life history diversity of Chinook Salmon in the Central Valley of California, but here they investigate juvenile outmigration phenotypes, which have been little studied in this system. By sampling juveniles over a 20+ year time frame as they out‐migrate through the San Francisco Estuary and genotyping those samples at thousands of SNPs, Thompson et al. (2024) discovered hidden biocomplexity in the system. They showed that there is both variation in outmigration timing among the four runs (spring, winter, late‐fall, and fall run), as well as variation among populations within spring run. Using their genomic assignments, they also showed how the current method of assigning individuals to run type based on length and outmigration timing was error prone and has led to overestimates of population sizes for the various ESA listed runs of Chinook Salmon. This study highlights the importance of correctly identifying and protecting this biocomplexity in order to promote a healthy and resilient portfolio of populations in the Central Valley, which is the only place in the entire species range where four runs co‐occur.

Miettinen et al. (2024) investigated whether fisheries practices for Atlantic Salmon (*S. salar*) in the northern Baltic Sea across a 93‐year period have led to harvest of specific stocks and age classes that have altered genetic variation associated with age at maturity. Subpopulations from the largest Baltic River complex (Tornio‐Kalix) were more frequent in early than late season catches within a year, indicating that timing of the fishery affected stock composition. They found that fishing early in the season preferentially targeted older maturing fish that also had the corresponding late allele at a known large effect gene (vgll3). However, the frequency of the late allele fluctuated considerably over the 93‐year period with a decrease from 1930s to 1970s, but then increased into early 2000s while remaining relatively stable in recent decades. Fisheries intensified in the 1960s through 1980s, which may have contributed to the decline of older, larger fish and corresponding decrease of the late allele during this period.

Carbonneau et al. (2024) investigated remote populations of Atlantic Salmon in the northernmost regions of North America to characterize genetic structure, variation in marine vs. estuarine life histories, and composition of stocks in the subsistence fishery in Koksoak estuary. Approximately 14 K SNP markers from genotyping by sequencing revealed hierarchical structure with isolation by distance among populations. While there was no apparent structure between marine vs. estuarine migrating fish, there were at least two candidate regions significantly associated with this trait. Finally, all fish harvested in the Indigenous fishery were from the nearest drainage (Koksoak) but included both migratory forms.

Kokkonen et al. (2024) used de novo transcriptomics data from 12 Cutthroat Trout (*O. clarkii*) subspecies and distinct lineages to aid in resolving taxonomic discrepancies among groups. Prior to widespread implementation of molecular tools to reconstruct phylogenies, 14 subspecies of Cutthroat Trout were recognized based on morphology, hydrology, and geological history. More recent studies have incorporated genetic data into phylogenetic studies though some relationships remained unresolved. To rectify obscure relationships in Cutthroat Trout groups, the authors used sequence data from 1983 gene transcripts. Their findings place Westslope Cutthroat Trout as the sister species to all other Cutthroat Trout lineages, contradicting some previous studies that place Coastal Cutthroat Trout as the immediate outgroup. Furthermore, their phylogeny reveals a potential gene transfer between Bonneville Cutthroat Trout and a population of Colorado River Green lineage Cutthroat Trout. This study illustrates the utility of transcriptomic data in resolving phylogenetic relationships of taxa with complicated and intertwined evolutionary histories.

Strait et al. (2024) examined effects of environment and introgression on cardiac performance of native populations of Westslope Cutthroat Trout that have experienced varying levels of hybridization with introduced Rainbow Trout. Since Rainbow Trout have higher thermal tolerance than Westslope Cutthroat Trout, it has been postulated that introgression between the two species may improve response of native Cutthroat Trout populations to climate change. In this study, they tested whether invasive hybridization with Rainbow Trout improved cardiac performance under heat stress, but did not find a significant difference in cardiac performance between fish with various levels of admixture. However, there was a significant effect of thermal regime on cardiac performance, suggesting that environmental factors were a more substantial factor in physiological performance than introgression.

### 2.3 Adaptive variation from whole‐genome resequencing studies

Several studies in this Special Issue investigated the crucial role of genomic diversity in maintaining life history variation and enabling natural populations to adapt to local environments. Three papers investigated genomic variation associated with locally adapted traits across populations (Lecomte et al., 2024; Narum et al., 2024; Willis et al., 2024), two others focused on genomic variation to adapt under scenarios of climate change (Tigano et al., 2024) or persist through ecological disturbance (Frei et al., 2024), and another on genomic differences between hatchery reared versus wild stocks (Howe et al., 2024).

Lecomte et al. (2024) examined SNPs, structural variants (SVs), and small indels between two populations of Atlantic Salmon in North America with differences in life history traits (i.e., age at smoltification and maturity). This was the only study in this Special Issue to collect both short‐read (~16× coverage per sample of Illumina paired‐end 150 base; NovaSeq6000) and long‐read (~20× coverage per sample of Oxford Nanopore; PromethION24) sequencing data for individual fish within a study. In this study, short‐read data provided variation at >8 million SNPs, while long‐read data enabled over 115,000 SVs and 1 million small indels to be identified across the genome. Outlier analyses identified putatively adaptive genomic variation between populations and candidate regions for further validation in future studies. These data provide an important resource for future studies.

Narum et al. (2024) synthesized multiple studies investigating genomic variation associated with adult migration timing in Steelhead and Chinook Salmon. A conserved genomic region of major effect (GREB1L/ROCK1) has been demonstrated to be highly associated with adult migration timing in both species. Subsequent validation studies with candidate SNP markers from this genomic region have revealed large effect sizes of candidate SNPs on multiple aspects of adult migration phenology such as passage timing after freshwater entry (Steelhead = 7.5–36.0%; Chinook Salmon = 27.9–47.6%) and arrival timing for spawning (Steelhead = 8.4–43%; Chinook Salmon = 4.7–79.9%). Studies further demonstrated distinct patterns of phenotypic association and linkage disequilibrium within and among lineages of each species. This study also discusses conservation implications of this major effect region for Columbia River lineages of each species, and future directions of research.

Willis et al. (2024) investigated age at maturity and iteroparity in Steelhead, two traits believed to be linked to adaptive resilience in natural populations. Despite their historical prevalence, repeat‐spawning individuals are rare with low return rates in the Columbia River basin. Out‐migrating adults exhibit different maturation phenologies, either consecutive or skip spawning. Using low‐coverage whole‐genome sequencing, the study identified genomic regions associated with iteroparity phenotypes. Notably, a significant region on chromosome 25 contains two genes (estradiol receptor beta, “ERβ”; and glycoprotein hormone beta‐5, “GPHB5”), involved in estrogen sensitivity and reproductive tissue expression in mammals. Allele frequencies from the ERβ and GPHB5 regions varied with age at maturity in females but not males. These genes also shared linkage disequilibrium with a gene on the same chromosome associated with age at maturity (sine oculis homeobox homolog 6, “SIX6”), suggesting overlapping genetic pathways governing phenology of age at maturity and iteroparity in Steelhead.

Tigano et al. (2024) examined the adaptive potential of Kokanee Salmon (*O. nerka*), a non‐migratory ecotype of Sockeye Salmon, in the face of climate change. By integrating analyses of genetic variation, genotype–environment associations, and climate modeling based on genomic data from 224 whole genomes sampled from 22 lakes in Canada, they found that extreme temperatures, particularly warmer temperatures, had the strongest signature of selection in the genome and were the best predictors of adaptive genomic variation. Genomic offset estimates, a measure of climate vulnerability, were correlated with increases in extreme warm temperatures, indicating a risk of future heat waves. However, levels of standing genetic variation were not correlated with genomic offset. The study by Tigano et al. (2024) thus emphasizes the importance of integrating different sources of information and genomic data to predict the vulnerability of populations and species to climate change accurately.

The study by Frei et al. (2024) investigated the impact of environmental change on genomic diversity and its consequences for species survival. Using whole‐genome resequencing data from four species of Lake Constance Alpine Whitefish (*Coregonus gutturosus*, *C. arenicolus*, *C. macrophthalmus*, *C. wartmanni*), spanning a period of anthropogenic disturbance, the researchers tracked changes in genomic diversity over time. They found a significant reduction in genomic diversity during the disturbance period, which has not yet recovered. This reduction ranged from 18% to 30% across species. Moreover, they observed a homogenization of interspecific allele frequency differences in potentially ecologically relevant genes, indicating the loss of adaptive genomic differentiation between species. The rapid erosion of genomic variation has occurred in combination with the loss of adaptive differentiation that evolved over thousands of years, and this finding underscores the vulnerability of biodiversity in young adaptive radiations to environmental disturbance. The study emphasizes the importance of historical collections in documenting biodiversity loss and underscores the need for effective global biodiversity conservation efforts.

Howe et al. (2024) examined genomic differences between three pairs of hatchery‐reared versus wild stocks of Chinook Salmon from Alaska. Over 6 million SNPs from low‐coverage whole‐genome sequencing were tested for differences and 14 outlier peaks were detected with high genetic differentiation between hatchery–wild pairs. However, none of the outlier peaks were shared across the three comparisons, suggesting that genetic differences among sample collections were population specific. The potential mechanisms for differences between pairs were unknown, but the authors suggested that body size at release, maturation timing, or environmental differences in hatchery rearing locations could have led to genomic differences. While pairs of sample collections in this study varied by multiple decades to test for differentiation over a few generations, further studies with hatchery–wild pairs with temporal replication would offer insight into whether genomic differences are consistent within each system.

## 3 FUTURE DIRECTIONS

The collection of articles within this Special Issue showcases some of the many advances in salmonid genetics and demonstrates how the field is rapidly advancing. This progress is set to continue as sequencing technology and computational methods further advance. Together, they clearly indicate that the continued application of genetic methods holds immense promise for understanding the genetic basis of key traits, enhancing breeding programs, and conserving genetic diversity for the future. Looking ahead, we would like to highlight a few areas that provide particularly promising future research directions in salmonids.

First, long‐term field studies are essential for unraveling the complex interplay between genetics and environmental factors that influence contemporary populations. This is because long‐term studies can provide invaluable insights into the adaptive potential of species in a natural setting and their resilience to multiple environmental changes over time. Moreover, longitudinal data enable the identification of genetic variants associated with key adaptive traits and their stability over time, laying the foundation for informed conservation strategies and sustainable management practices. We think that the generation of such long‐term datasets should continue, and that these datasets will be important in gaining insights into the detailed processes that modulate salmonid populations in the wild.

Second, baseline data, particularly genomic information derived from historic samples, are crucial for assessing changes in population structure, and genetic diversity. Museum collections offer a treasure trove of historical samples (otoliths, scales, etc.) that can span several decades, providing a unique opportunity to reconstruct historical genetic variation and track changes in allele frequencies over time. Leveraging these collections can enrich our understanding of past genetic structure and diversity to inform conservation and management efforts (e.g., genetic rescue), such as changes in the genomic architecture of life history traits (Besnier et al., 2024).

Third, adopting long‐read and or high coverage whole‐genome sequencing technologies is essential for identifying structural genomic variation including insertions, deletions, and chromosomal inversions, which play pivotal roles in shaping genomic architecture and driving evolutionary innovation (e.g., Mérot et al., 2020; Wellenreuther et al., 2019). With these sequencing approaches, researchers can create catalogues of genomic variants, ranging from SNPs to larger‐scale chromosomal rearrangements, to move beyond pure SNP‐based analyses. Increasing evidence shows that structural variants are ubiquitous and underpin phenotypic diversity and adaptive evolution in salmonids, and the greater incorporation of structural variants in future studies has the potential to reveal new insights into the genomic architecture of keystone traits in salmonids.

Fourth, in parallel with increased genomic data acquisition, extensive phenotyping tools are needed to reveal the complex genotype–phenotype relationships of various traits in salmonids. High‐throughput tools have enabled the community to produce massive genetic and genomic data sets, but phenotypic information is often trailing behind relative to the genetic data. Salmonid‐specific phenotyping tools are on the rise (e.g., Tuckey et al., 2022), but the availability of tools is still a limiting factor. Integrating high‐throughput digital phenotyping approaches with genomic analyses enables the comprehensive characterization of phenotypic traits and variation between them, and this can help to uncover the genetic basis of traits and their co‐variance structure. Advances in tagging approaches have also led to improvements in tracking fish physiology and behavior (e.g., body temperature and heart rate, e.g., Svendsen et al., 2021; movement and migration patterns, e.g., Willis et al., 2020) that allow studies to investigate genetic and environmental drivers of these basic activities. Growth and aging techniques have been widely available for salmonids for decades and occasionally combined with genetic markers for age at maturity (e.g., Czorlich et al., 2018), but these phenotypes have not been routinely analyzed with genomic data to investigate patterns of association in most salmonid species. Several other traits remain as cryptic phenotypes, yet undiscovered in salmonid populations that occupy aquatic habitats with limited direct observation. Such phenotypic data are also indispensable for enhancing the accuracy of genomic predictions and accelerating the breeding of improved genetic lines with desirable traits in aquaculture and reintroduction programs.

Fifth, genetic data provide a crucial backdrop to inform the selection of individuals for the cryopreservation of sperm, representing a promising future strategy to biobank genetic diversity and to preserve valuable germplasm for future generations. This is important both from an aquaculture breeding practice perspective, where genetic diversity can be maintained through the storage of cryopreserved sperm, and from a conservation practice perspective, where local population genetics can be preserved for enhancement and restocking efforts (e.g., Wylie et al., 2023). By banking sperm from diverse individuals representing various populations and ecotypes, researchers can safeguard genetic resources against loss due to environmental disturbances, habitat degradation, or disease outbreaks.

## CONFLICT OF INTEREST STATEMENT

The authors state no conflict of interest.

## Data Availability

No data for this editorial article that provides an introduction to the Special Issue on Advances in Salmonid Genetics.
